# Determination of Ferret Enteric Coronavirus Genome in Laboratory Ferrets

**DOI:** 10.3201/eid2309.160215

**Published:** 2017-09

**Authors:** Tian-Cheng Li, Sayaka Yoshizaki, Michiyo Kataoka, Yen Hai Doan, Yasushi Ami, Yuriko Suzaki, Tomofumi Nakamura, Naokazu Takeda, Takaji Wakita

**Affiliations:** National Institute of Infectious Diseases, Tokyo, Japan (T.-C. Li, S. Yoshizaki, M. Kataoka, Y.H. Doan, Y. Ami, Y. Suzaki, T. Wakita);; Research Foundation for Microbial Diseases of Osaka University, Kagawa, Japan (T. Nakamura);; Osaka University, Osaka, Japan (N. Takeda)

**Keywords:** Ferret enteric coronavirus, ferret systemic coronavirus, coronavirus, next-generation sequencing, ferret, genome, viruses, enteric infections

## Abstract

Ferret enteric coronavirus (FRECV) RNA was detected in laboratory ferrets. Analysis of the complete genome sequence of 2 strains, FRCoV4370 and FRCoV063, revealed that FRECV shared 49.9%–68.9% nucleotide sequence identity with known coronaviruses. These results suggest that FRECV might be classified as a new species in the genus *Alphacoronavirus.*

Ferret coronavirus (FRCoV), a novel animal coronavirus (CoV), was identified in ferrets (*Mustela putorius furo*) in 2006 (*1*). However, only partial sequences of FRCoV have been analyzed, including portions of open reading frame (ORF) 1b and the full-length spike protein (S), nonstructural protein 3c (3c), envelope protein (E), membrane protein (M), nucleocapsid protein (N), and accessory genes (3x and 7b), and only in 3 strains: 1 ferret systemic coronavirus (FRSCV), FRSCV MSU-1 strain (GenBank accession no. GU338456); and 2 ferret enteric coronaviruses (FRECVs), FRECV MSU-2 strain (GenBank accession no. GU338457) and FRECV No22 strain (GenBank accession no. LC029419) (*2**,**3*). Genetic analyses based on these partial sequences showed that the ferret coronavirus is closer to mink coronavirus (MCoV) than to other CoVs and appears to be a member of the genus *Alphacoronavirus* in the subfamily *Coronovirinae*, which also contains the genera *Betacoronavirus* and *Gammacoronavirus* (*4*). To further understand the constellation of FRCoVs, we analyzed the complete genome.

## The Study

In our previous study, we detected ferret hepatitis E virus (HEV) in fecal samples from laboratory ferrets and confirmed that 40 (63.5%) of the 63 ferrets were infected with ferret HEV (*5*). For the observation of the ferret HEV particles by electron microscopy, we used a 10% suspension prepared from the fecal specimen from 1 of the ferrets (no. F4370), concentrated by ultracentrifugation and purified by sucrose gradient ultracentrifugation. However, instead of finding ferret HEV particles, we observed many coronavirus-like particles in fractions 4, 5, and 6, with densities of 1.230 g/cm^3^, 1.214 g/cm^3^, and 1.198 g/cm^3^ (data not shown). These particles ranged from 60 nm to 120 nm in diameter; most of them had a spike structure, suggesting that the ferrets were infected with a CoV-like virus.

To precisely examine these CoV-like particles, we diluted 63 fecal specimens with 10-mmol/L PBS for preparation of the 10% suspension. We extracted the RNA by using a MagNA Pure LC Total Nucleic Acid isolation kit (Roche Applied Science, Mannheim, Germany) according to the manufacturer’s recommendations. We performed an FRSCV-specific reverse transcription PCR (RT-PCR) with the primer set G1F (5′-CTGGTGTTTGTGCAACATCTAC-3′) and G1R (5′-TCTATTTGCACAAAATCAGACA-3′) and an FRECV-specific RT-PCR with the primer set G2F (5′-GGCATTTGTTTTGATAACGTTG-3′) and G2R (5′-CTATTAATTCGCACGAAATCTGC-3′) (*2*).

RT-PCR results revealed that, of the 63 ferret fecal specimens, 22 (34.9%) were positive for FRSCV RNA, 53 (84.1%) were positive for FRECV RNA, and 15 (23.8%) were positive for both FRSCV and FRECV RNA. The specimen from ferret F4370 was positive only for FRECV RNA. These results indicated that the ferrets were infected extensively with FRCoV. However, we observed no signs such as weight loss or diarrhea in the ferrets.

We extracted RNA from a pool of fractions 4, 5, and 6 from the F4370 fecal sample suspension and analyzed the complete genome sequence of FRCoV by using a next-generation sequence analysis (*6*). FRCoV4370 (GenBank accession no. LC119077) has a genome size, gene order, genomic organization, and structure similar to those of known alphacoronaviruses. The complete genome of FRCoV4370 contains 28,525 nt and a poly (A) tail. Except for the 5′-terminus 261 nt and 3′-terminus 247 nt untranslated regions, FRCoV4370 encodes 9 proteins: ORF1a, ORF1a/1b, S, 3c, E, M, N, 3x, and 7b (Table). Although the ORF7a protein was found in the MCoVs WD1127 (GenBank accession no. HM245925) and WD1133 (GenBank accession no. HM245926), we did not find it in FRCoV4370.

**Table:** Nucleotide positions of proteins of FrCoVs from analysis of complete virus genome and number of amino acids compared with MCoVs*

Proteins	Nucleotide position			No. amino acids						
	FRCoV4370	FRCoV063		FRCoV4370	FRCoV063	MSU-2	MSU-1	No22	WD1127	WD1133
ORF1a	262–12228	262–12231		3,988	3,989	NA	NA	NA	4,018	4,006
ORF1a/b	262–20222	262–20225		6,653	6,554	NA	NA	NA	6,682	6,670
Spike	20215–24564	20218–24567		1,449	1,449	1,449	1,457	1,435	1,438	1,429
ORF3c	24585–25328	24585–25328		247	247	247	83	247	247	69
E	25297–25545	25297–25545		82	82	82	82	82	82	82
M	25560–26351	25560–26363		263	267	263	263	265	268	268
N	26368–27492	26380–27504		374	374	374	374	374	376	376
ORF7a				NI	NI	NI	NI	NI	98	98
ORF3x	27501–27725	27514–27738		74	74	74	14	74	73	73
ORF7b	27664–28278	27677–28291		204	204	204	204	202	204	204

*E, envelope protein; FrCOV, ferret coronavirus; M, membrane protein; MCoV, mink coronavirus; N, nucleocapsid protein; NI, protein not identified; NA, sequence not available; ORF, open reading frame.

The complete genome of FRCoV4370 shared 49.9%–68.9% nucleotide identity with other known CoVs. Phylogenetic trees based on the complete genome demonstrated that FRCoV4370 is closer to MCoVs than to other CoVs, although it clearly separated into a distinct cluster (Figure, panel A). We observed similar phylogenetic clustering when we compared ≈8,300 nt sequences of the 3′-termini of CoVs. FRCoV4370 forms a new cluster with FRSCV MSU-1, FRECV MSU-2, and No22, being separated from the cluster formed by MCoVs (Figure panel B) In addition, BLAST analysis (http://blast.ncbi.nlm.nih.gov/Blast.cgi) using the nucleotide sequences described previously revealed that FRCoV4370 shared 94% identity with FRECV MSU-2, which is higher than that of FRSCV MSU-1 (89%), indicating that FRCoV4370 belongs to the FRECVs.

**Figure:**
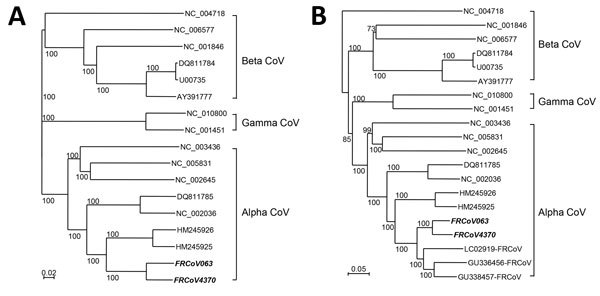
Phylogenetic relationships between ferret coronaviruses (FrCoVs, shown in bold italics) and other known coronaviruses (CoVs). A) Complete genome; B) partial 3′-terminus genome. The nucleic acid sequence alignment was performed using ClustalX version 1.81 (http://www.clustal.org). The genetic distance was calculated by Kimura’s 2-parameter method. Phylogenetic trees with 1,000 bootstrap replicates were generated by the neighbor-joining method (Njplot 2.3, http://njplot.sharewarejunction.com/). Comparison CoVs identified by GenBank accession number. Scale bars indicate substitutions per site.

As is the case for other CoVs, 2 long ORFs were predicted in the FRCoV4370 genome: ORF1a contains 11,967 nt, from nt 262 to 12228, encoding 3,988 aa; ORF1b encodes 7,830 nt, from nt 12393 to 20222. The coronaviruses have a pseudoknot tertiary structure that allows a ribosomal shift of the reading frame between ORF1a and ORF1b (*7**–**9*). We also found the slippery sequence for the ribosomal shift (UUUAAAC) in the FRCoV4370 genome at nt positions 12192–12198. The ribosomal shift may have resulted in generation of the ORF1a/b protein encoding 19,962 nt and the deduced 6,653 aa.

We identified 4 structural proteins in FRCoV4370: S, E, M, and N. The S protein is the largest, consisting of 1,449 aa, which shared 92.5% aa identity with FRECV MSU-2, 81.4% with FRSCV MSU-1, and 84.5% with FRECV No22. The BLAST analysis of the S protein showed that it had low aa identities (43%–66%) with other CoVs. Because the S protein is a major inducer of virus-neutralizing antibodies, the antigenicity and serotype of FRCoVs might be different from those of other CoVs.

The E protein, the smallest structural protein, encodes 82 aa (which is the same number encoded by other known FRCoVs) and shared 94.8% aa identity with FRECV MSU-2, 85.6% with FRSCV MSU-1, and 86.7% with FRECV No22. The 263-aa M protein shared 81.6% aa identity with FRECV MSU-2. The low aa identity between the FRECV strains suggests that the membrane gene is highly variable. The N protein of FRCoV4370 contains 374 aa, making it the shortest among the alphacoronaviruses. The N protein shared 96.3% aa identity with FRECV MSU-2, 93.4% with FRSCV MSU-1, and 80.2% with FRECV No22.

ORF3c was identified in FRCoV4370, as observed in MCoVs. This protein is an accessory triple-spanning membrane protein and is analogous to SARS-CoV 3a protein (*10*). The predicted ORF3c protein contains 247 aa and shared 96.8% aa identity with FRECV MSU-2 and 77.6% with FRECV No22. The 2 other nonstructural proteins, 3x and 7b, are located downstream of the N protein and encode 74 aa and 204 aa, respectively.

We also detected FRECV RNA in the fecal specimens of 9 ferrets from another farm in the United States and analyzed the complete genome of 1 strain, FRCoV063 (LC215971). The genome of FRCoV063 shared 94.0% nucleotide identity with FRCoV4370 and had a similar structure (Table; Figure), suggesting that ferret coronavirus infection is common in ferrets, and genetically similar FRCoV strains circulate at the ferret farms.

## Conclusions

Since the initial identification of FRCoV in ferrets in 2006, many sequences of FRSCV and FRECV strains have been analyzed (*1**–**3**,**11**–**15*). However, the complete genome sequences have not been determined and added to the public databases. In this study, we successfully analyzed the complete genome of 2 strains of FRECV, FRCoV4370 and FRCoV063, and found that they shared 94.0% nucleotide identity with each other but 49.9%–68.9% nucleotide identities with other known CoVs, suggesting that the ferret coronaviruses might be classified as a new species in the genus *Alphacoronavirus*. This new knowledge of the complete genome sequence of FRECV will contribute to investigations of the diversity of animal CoVs and will help establish new taxonomic units.
